# Neuroimaging Correlates of Post-COVID-19 Symptoms: A Functional MRI Approach

**DOI:** 10.3390/diagnostics14192180

**Published:** 2024-09-29

**Authors:** Marine M. Tanashyan, Polina I. Kuznetsova, Sofya N. Morozova, Vladislav A. Annushkin, Anton A. Raskurazhev

**Affiliations:** 1 1st Neurological Department, Research Center of Neurology, 125367 Moscow, Russiaannushkin@neurology.ru (V.A.A.); raskurazhev@neurology.ru (A.A.R.); 2 Radiology Department, Research Center of Neurology, 125367 Moscow, Russia

**Keywords:** fMRI, COVID-19, post-COVID fatigue, cognitive impairment

## Abstract

Backgrounds and Purpose: Post-COVID syndrome is characterized by persistent symptoms, including fatigue and cognitive impairment. These symptoms may be experienced by up to 80% of patients. We aimed to identify possible patterns of brain activation underlying post-COVID fatigue. Methods: The study used functional MRI (Siemens MAGNETOM Prisma 3T scanner with a specially created protocol) of the brain in 30 patients with post-COVID fatigue syndrome and 20 healthy volunteers. Task functional MRI (fMRI) was performed using a cognitive paradigm (modified Stroop test). Eligible patients included adults aged 18–50 years with a >12 weeks before enrolment (less than 12 months) prior history of documented COVID-19 with symptoms of fatigue not attributable to any other cause, and with MFI-20 score > 30 and MoCA at first visit. Healthy control participants had no prior history of COVID-19 and negative tests for severe acute coronavirus respiratory syndrome with MFI-20 score < 30 and MoCA at first visit. Task fMRI data were processed using the SPM12 software package based on MATLAB R2022a. Results: Cognitive task fMRI analysis showed significantly higher activation in the post-COVID group versus healthy volunteers’ group. Between-group analysis showed significant activation differences. Using a threshold of T > 3 we identified eight clusters of statistically significant activation: supramarginal gyri, posterior cingulate cortex, opercular parts of precentral gyri and cerebellum posterior lobe bilaterally. Conclusions: Post-COVID fatigue syndrome associated with subjective cognitive impairment could show changes in brain functional activity in the areas connected with information processing speed and quality.

## 1. Introduction

Post-COVID syndrome (or long-COVID as it is frequently named) remains an elusive medical entity—despite the fact that the search term «post-COVID» reaches more than 36,000 results in a simple search of PubMed. According to WHO, it is defined «as the continuation or development of new symptoms 3 months after the initial SARS-CoV-2 infection, with these symptoms lasting for at least 2 months with no other explanation» [1]. One of the most persistent symptoms of this condition is fatigue and cognitive impairment, which may be present in up to 80% of patients [2]. Neurological symptoms, including anosmia, ageusia, fatigue, and cognitive impairment, have been frequently reported in patients manifesting COVID-19 sequelae [3,4].

These symptoms significantly reduce the quality of the patient’s life and lead to social and economic consequences [5]. Despite the growing volume of clinical data, the mechanisms underlying cognitive impairment in post-COVID syndrome remain understudied.

One of the promising methods of investigating pathophysiological changes in the brain is functional magnetic resonance imaging (fMRI), which allows assessment of the functional brain properties, including activation, deactivation and connectivity of various brain structures [6]. FMRI is a neuroimaging technique that has revolutionized the field of neuroscience by allowing researchers to non-invasively study brain function in vivo. The underlying principle of fMRI is based on the detection of changes in blood oxygenation and flow in response to neural activity. When a specific brain region is active, it requires an increased supply of oxygen and nutrients, leading to an increased blood flow and a higher concentration of oxygenated hemoglobin relative to deoxygenated hemoglobin. FMRI detects these changes through the Blood Oxygen Level Dependent (BOLD) contrast, which provides imaging areas of higher brain activity. This process, known as neurovascular coupling, forms the basis of fMRI signal detection. In a typical task fMRI experiment, participants are asked to perform specific tasks of different modalities, including cognitive, while lying in the MRI scanner. The tasks can vary widely depending on the research question, ranging from simple sensory stimulation or movement to complex decision-making paradigms. During the task, the MRI scanner acquires a series of images that capture changes in blood oxygenation levels across different brain regions [7].

Early studies using fMRI revealed changes in functional activity and connectivity in several key neural networks, including the brain’s passive mode network (default mode network), the frontoparietal network, and the executive network. Several authors have observed that COVID-19 can cause changes in brain activity and connectivity [8,9]. Appelt et al. showed that cognitive functions decreased after a coronavirus infection, which was confirmed by electrophysiological data [10]. These changes may underlie the reported cognitive impairments, fatigue, including decreased attention, decreasing memory, and difficulty in making decisions [11].

The aim of this study was to study post-COVID syndrome brain functional activity using fMRI.

## 2. Materials and Methods

### 2.1. Study Population and Clinical Data

This was a cross-sectional, observational study, using functional MRI of the brain in 30 patients with post-COVID fatigue syndrome and 20 healthy volunteers. Eligible patients included adults aged 18–50 years with a >12 weeks before enrolment (less than 12 months) prior history of documented COVID-19 with symptoms of fatigue not attributable to any other cause, and with MFI-20 (Multidimensional Fatigue Inventory) score > 30 and MoCA at first visit. Healthy control participants had no prior history of COVID-19 and negative tests for severe acute coronavirus respiratory syndrome with MFI-20 score < 30 and MoCA at first visit.

### 2.2. MRI Data Acquisition and Processing

Data were acquired from 30 patients with post-COVID fatigue syndrome and 20 healthy volunteers using Siemens MAGNETOM Prisma 3T scanner (München, Germany) with a specially created protocol. Conventional MRI sequences (i.e., axial T2 (Acquisition parameters (AP):TR = 5500 ms, TE = 106 ms, FOV = 220 mm, slice thickness = 3 mm, in-plane resolution 0.7 × 0.7 mm, distfactor 30%, number of slices = 35, acquisition time = 2 min 14 s) and T2FLAIR (AP: TR = 9000 ms, TE = 88 ms, FOV = 220 mm, slice thickness = 4 mm, in-plane resolution 0.4 × 0.4 mm, number of slices = 27, distfactor 30%, acquisition time = 1 min 50 s) sequences and three-dimensional gradient echo sagittal T1 sequence (AP: TR = 2300 ms, TE = 2.98 ms, FOV = 256 mm, slice thickness = 1 mm, in-plane resolution 1 × 1 mm, number of slices = 176, acquisition time = 5 min 12 s)) were used to make sure that both post-COVID patients and healthy volunteers did not have serious brain pathology (e.g., post-stroke lesions, neoplasms, demyelination), otherwise they were excluded from the study. Three-dimensional gradient echo sagittal T1 sequence was also used as structural data for functional MRI (fMRI) post-processing. Imaging parameters were the same as in our previous work [12]. Consequently, three healthy volunteers were excluded from the subsequent analysis because of presence of lacunes in cerebral white matter.

Task fMRI was performed using a cognitive paradigm (modified Stroop test) with the same parameters as in our previous study and consisted of 120 brain volume measurements organized in 4 baseline blocks of 15 measurements and 4 active (task performance) blocks of 15 measurements, each block duration of 30 s (AP: TR 2000 ms, TE 30 ms, slice thickness 2 mm, matrix 190 × 190 mm, flip angle 70 degrees, distance factor 10%, number of slices 66, acquisition time = 4 min 12 s). During the baseline blocks, the MRI compatible screen displayed a white cross on the black background; during the task performance blocks, the MRI compatible screen displayed differently colored names on the black background. If the font color and the written color name coincided (e.g., «green» was written in green) the patients and healthy subjects had to make a mental note of it for themselves and vice versa: if there was color and meaning mismatch, they were instructed to ignore it. All patients and healthy subjects were trained before examination outside the scanner.

SPM12 software package for MATLAB was used for task fMRI data processing. The standard preprocessing algorithm included spatial realigning, co-registration, normalization and smoothing of functional data using a Gaussian filter with full width at half maximum (FWHM) kernel size of 8 mm. First-level general linear model (GLM) analysis of functional data was performed for each subject separately with the following grouping of patients and healthy volunteers receiving color maps of activation superimposed on anatomical data and a detailed statistical report including level of statistical significance, volume and coordinates of activation area in the MNI stereotaxic space [13] for each group (one-sample t-test with *p* ≤ 0.05, the effect size of multiple comparisons assessed by family-wise error (FWE), T > 4). Afterwards two-sample t-test was used for between-group analysis with a statistical significance threshold of *p* ≤ 0.001 without correction for multiple comparisons, T > 3. Areas of interest localization by Brodmann fields was performed using xjView 9.0 toolbox for SPM12 (Human Neuroimaging Lab, Baylor College of Medicine), which provided a detailed pictorial and statistical report of the experimental data. Presented figures were created by uploading and viewing the received results in MRIcroGL software v1.2.20220720 (nitrc.org). Descriptive statistics were performed in R (version 4.4.1) via RStudio environment (version 2024.04.2), utilizing the following packages: ‘tidyverse’, ‘gtsummary’. Between-group comparisons were made using the Wilcoxon rank sum test (in case of continuous variables) or Pearson’s Chi-squared test (for categorical variables). All test were two-sided, with alpha-level <0.05.

## 3. Results

Participant demographics and clinical data are listed in Table 1.

**Figure 1:**
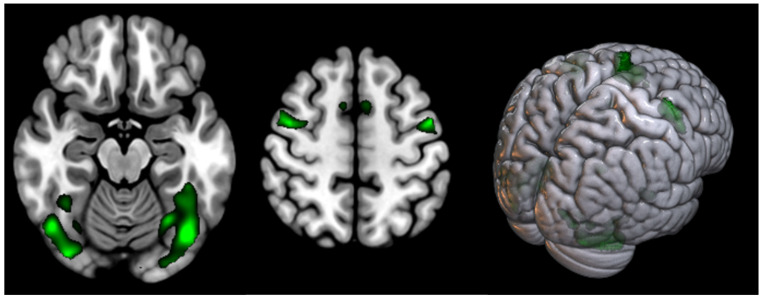
Areas of cortical activation in healthy volunteers during modified Stroop test performance (shown in green).

Received data analysis in the healthy volunteers group showed significant activation in the supplementary motor area, premotor and visual cortex bilaterally (Table 2). Activation clusters are shown in green (Figure 1).

**Figure 2:**
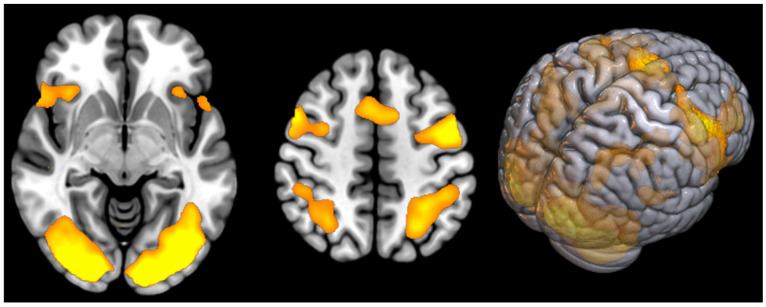
Areas of cortical activation in post-COVID patients during modified Stroop test performance (shown in yellow).

Received data analysis in the post-COVID patients group showed significant activation in the supplementary motor area, premotor, visual cortex and superior parietal lobules bilaterally (Table 3). Activation clusters are shown in yellow (Figure 2).

**Figure 3:**
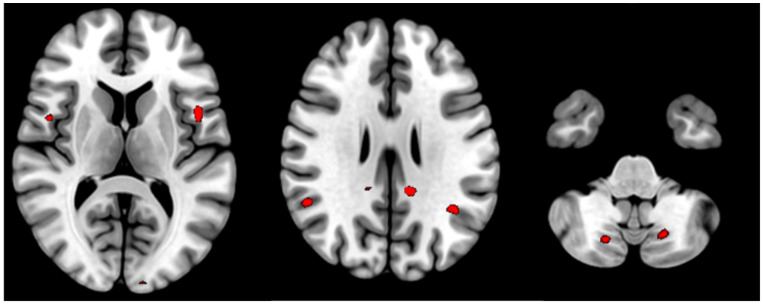
Results of between-group analysis. Clusters of greater activation in patients’ group are shown in red.

Between-group analysis showed significant activation differences. Patients had higher activation in supramarginal gyri, posterior cingulate cortex, opercular parts of precentral gyri and cerebellum posterior lobe bilaterally (Table 4). Regions with activation differences are shown in red (patients more than healthy controls) on Figure 3.

## 4. Discussion

Brain changes have been repeatedly reported since the onset of SARS-CoV-2 infection. However, limited literature exists on brain alterations in post-COVID syndrome, a condition associated with cognitive impairment and fatigue, albeit that collecting this information is of vital importance for subsequent investigation of therapy effectiveness. As shown by Díez-Cirarda et al., post-COVID syndrome patients presented with functional connectivity changes, characterized by hypoconnectivity between left and right para-hippocampal areas, and between bilateral orbitofrontal and cerebellar areas, compared to controls which can explain structural and functional brain abnormalities after acute infection [14]. Chang et al. showed results in patients with post-COVID syndrome which demonstrated compensatory neural processes with greater usage of alternate brain regions, and reorganized networks, to maintain normal performance during working memory tasks [15]. Brain fog, characterized by cognitive impairment and fatigue, is a common post-COVID-19 symptom affecting 7.2% of patients [16]. This condition involves difficulties with concentration (being described as “thinking/focusing difficulty”), memory, and executive function [17].

The main findings of the present study are that cognitive task fMRI analysis showed significantly higher activation in the post-COVID group versus healthy volunteers’ group. Patients had higher activation in supramarginal gyri, posterior cingulate cortex, opercular parts of precentral gyri and cerebellum posterior lobe bilaterally.

Research suggests that the posterior cingulate cortex (PCC) plays a crucial role in fatigue-related neural processes. Multiple studies using magnetoencephalography (MEG) have identified PCC activation during self-evaluation of physical and mental fatigue, as shown in our study [18]. Furthermore, increased cerebral blood flow in the PCC has been observed in cancer patients experiencing treatment-related fatigue [19]. Research on the supramarginal gyrus (SMG) reveals its diverse roles in cognitive processes. The left SMG is involved in phonological processing during reading [20]. The right SMG plays a crucial role in temporal perception, as stimulation of this area alters time measurement [21]. Additionally, a large cluster, including the anterior ventral premotor cortex, insula, and postcentral gyrus, has been identified as critical for supraspinal fatigue following exhausting aerobic exercise [22]. These findings highlight the SMG’s importance in various cognitive functions, including phonological processing, temporal perception, and fatigue-related processes.

Opercular part of precentral gyrus turns out to be an «agranular» region lacking any distinct layer 4, a trait typical of motor regions, and seems to be dedicated to more local operations, of the finite state/linear adjacency kind, probably being the third sub-operation of linearization to that part of Broca’s area [23]. This could obviously be connected with paradigm task execution demanding high-speed understanding and processing of linguistic information, which requires involving more resources in post-COVID patients with brain fog.

Recent studies have highlighted the cerebellum’s role in fatigue perception and motor learning. Sleep deprivation alters functional connectivity patterns in the cerebellum posterior lobe [24]. Sleep quality has a deep impact on mental health and emotional well-being. Sufficient and restful sleep is crucial for cognitive functioning, memory consolidation, and emotional regulation [25]. Sleep disturbances, such as insomnia, hypersomnia, and parasomnias, have become increasingly prevalent during the COVID-19 pandemic [26]. Perhaps one of the reasons for the development of subjective fatigue is the effect of sleep disorders associated with coronavirus infection; however, in our study we did not evaluate sleep disorders and did not conduct a separate assessment of these conditions.

Decreased cerebellar excitability correlates with reduced physical fatigue perception but impaired motor control [27]. These findings collectively suggest that the cerebellum plays a significant role in regulating fatigue perception, motor learning, and adaptation. Fatigue-related processes may compete with performance-related processes for cerebellar resources, highlighting the complex interplay between fatigue, motor control, and cerebellar function.

Based on abovementioned results of this and previous studies, it could be assumed that observed differences occurred in the areas strongly connected with information processing and fatigue perception.

However, our observations in these post-COVID cases demonstrated that the brain fog phenomenon is a subjective condition with no clinically detected cognitive impairment (in both groups, the average MoCA scores were 28).

The MFI 20 scale is a widely used tool for assessing fatigue levels and declining quality of life in patients with various diseases [28]. One of the pathophysiological mechanisms of post-COVID fatigue is disruption of post-exhaustion corticomotor inhibition GABA-ergic dysfunction, defined as a debilitating, non-transient feeling of physical and mental tiredness or exhaustion characterized by lack of energy, muscle weakness and deficit in concentration [29]. In our study, we found that patients with post-COVID syndrome have higher scores on this scale, indicating that they experienced more pronounced symptoms of fatigue and decreased quality of life compared to healthy volunteers. These results confirm growing research indicating that post-COVID syndrome can have serious health consequences, including chronic fatigue. Our findings highlight the need for further research to elucidate the mechanisms underlying chronic fatigue in post-COVID syndrome, as well as to find effective treatments for this condition.

The techniques based on research using fMRI have significant drawbacks, one of which is that, like all hemodynamic-based modalities, it measures a surrogate signal whose spatial specificity and temporal response are subject to both physical and biological constraints. A more important shortcoming is that this surrogate signal reflects neuronal mass activity [30].

However, fMRI studies have been providing researchers with a great amount of valuable neurobiological information for many years, and the use of fMRI in the study of post-COVID syndrome has the potential to significantly enhance our understanding of the neurobiological impact of COVID-19, guide the development of diagnostic and prognostic tools, and inform the creation of targeted, effective treatments for patients suffering from lingering effects of the disease.

## 5. Conclusions

Post-COVID fatigue syndrome associated with subjective cognitive impairment could show changes in brain functional activity in the areas connected with information processing speed and quality.

## 6. Limitations

The study has a number of limitations, of which the most important are a small sample size, potential for selection bias, vague definitions of post-COVID syndrome and fatigue. The pilot study’s small sample size and lack of an external control group may limit the generalizability of the findings. This is a single center study, which let us consider it as a pilot, requiring future research including larger numbers of patients. Recruiting a sufficiently small and homogeneous sample of post-COVID patients limits the generalizability of findings. The symptoms of post-COVID fatigue can fluctuate over time, complicating the interpretation of fMRI data collected at a single time point. COVID-19 can cause vascular and inflammatory changes that may affect cerebral blood flow independent of neural activity. Since fMRI relies on blood oxygen level-dependent (BOLD) signals, these changes can confound the interpretation of results.

## Figures and Tables

**Table 1:** Participant demographics and clinical data.

Characteristic	Control *N* = 20 ^1^	Post-COVID *N* = 30 ^1^	*p*-Value ^2^
Age	42 (37, 47)	34 (27, 50)	0.4
Gender			0.092
F	10 (50%)	22 (73%)	
M	10 (50%)	8 (27%)	
MoCA	28.00 (26.50, 29.00)	28.00 (27.00, 29.00)	0.7
MFI-20	23 (18, 28)	63 (54, 74)	<0.001

^1^ Median (Q1, Q3); n (%); ^2^ Wilcoxon rank sum test; Pearson’s Chi-squared test.

**Table 2:** Activation in the group of healthy control subjects.

	T	P*_FWEcorr_*	Number of Voxels	Peak MNI Coordinate
Supplementary motor cortex	11.98	<0.000	799	0 0 68
Occipital cortex (Fusiform gyrus) (L)	10.34	<0.001	1507	60 8 30
Occipital cortex (Fusiform gyrus) (R)	9.24	<0.005	706	44 −66 −20
Precentral gyrus (L) (BA6)		<0.007	608	−54 −2 48
Precentral gyrus (R) (BA6)	8.67	<0.009	221	42 −2 56

**Table 3:** Cerebral activation areas in the group of post-COVID patients with fatigue syndrome.

	T	P*_FWEcorr_*	Number of Voxels	Peak MNI Coordinate
Supplementary motor cortex	10.98	<0.000	1002	2 8 58
Occipital cortex (Fusiform gyrus) and Cerebellum Posterior Lobe (L + R)	16.96	<0.000	13,405	−26 −86 −16
Precentral gyrus (L) (BA6)	14.38	<0.000	1739	−48 −2 46
Precentral gyrus (R) (BA6)	10.73	<0.000	2186	52 14 44
Parietal Superior Lobule (L) (BA7)	9.21	<0.000	1066	−24 −60 46
Parietal Superior Lobule (R) (BA7)	8.43	<0.000	315	28 −58 46

**Table 4:** Between-group analysis of activation differences (patients more than healthy controls).

	T	P*_uncorr_*	Number of Voxels	Peak MNI Coordinate
Posterior cingulate cortex (L)	4.06	<0.000	14	−12 −42 28
Posterior cingulate cortex (R)	3.32	0.001	2	6 −44 50
Supramarginal gyrus (L)	3.37	<0.000	23	−38 −54 30
Supramarginal gyrus (R)	3.49	<0.000	22	52 −50 22
Opercular part of Precentral gyrus (L)	3.77	<0.000	12	−46 8 8
Opercular part of Precentral gyrus (R)	3.47	0.001	11	48 4 10
Cerebellum Posterior Lobe (8) (L)	3.33	0.001	67	−18 −68 −42
Cerebellum Posterior Lobe (8) (R)	3.25	0.001	34	18 −70 44

## Data Availability

The data that support the findings of this study are available from the corresponding author upon reasonable request.
